# Statistical Reports on the Sickness, Mortality, and Invaliding among the Troops in the United Kingdom, the Mediterranean, and British America

**Published:** 1839

**Authors:** 


### Medico-Chirurgical Review,

### No. LXI.

[No. 21 of a Decennial Series.]

APRIL 1, to JULY 1, 1839.

### Statistical Reports on the Sickness, Mortality, and Invaliding among the Troops in the United Kingdom, the Mediterranean, and British America; Prepared from the Records of the Army Medical Department and War-Office Returns.

In the last Number but one of this Journal we noticed the Statistical Reports on the sickness and mortality of our Troops in the West Indies. We have now before us a similar series of facts and observations on the health of our brave soldiers in the United Kingdom, in the Mediterranean, and in British America.

We are informed by Captain Tulloch, by whom these Reports are drawn up, that they are the offspring of the same course of investigation, which was adopted in the case of the British Force in the West Indies. The Reports are as follows:—

1. On the Sickness, Mortality, and Invaliding among Troops serving in the United Kingdom.
2. Ditto, ditto, among those serving in the Mediterranean Stations.
3. Ditto, ditto, among those serving in British America.

And Captain Tulloch adds:—

"It has been deemed expedient to combine these in the present volume, because, as they serve to illustrate the influence of climates of which the diseases are in many respects similar, the reference which may become necessary for the purpose of comparison will thereby be materially facilitated.

In the preparation of these documents, particularly the medical details, I have been indebted to the assistance of the same parties to whom my obligations were expressed for similar aid in the West India Report, and the materials have principally been derived from the same source, viz., the Returns and Reports transmitted to the Director-General of the Army Medical Department by the Medical Officers in charge of the different Corps and Military Stations in each Command.

From these documents more ample details might have been given illustrative of the topography of the Mediterranean and American Colonies; but as information on that subject can be obtained from other sources already before the public, it has been deemed expedient to limit that portion of the Report to such points as are likely to have exerted a material influence on the health of the Troops.

Many interesting speculations in regard to the supposed causes of some diseases, and the contagious or non-contagious properties of others, might also have been supplied from the same source, but these being inconsistent with a Report of this nature, have only been adverted to where sufficient numerical evidence could be obtained for estimating their accuracy. Limited as that portion of the investigation has necessarily been, however, it will at least serve to develop facts which may prove useful in directing the future inquiries of others on the same subject." 4.

Were any person, unacquainted with the peculiar features of the British service, to be told that Reports were drawn up, and laid before the Parliament and the Public, on the sickness and on the diseases of troops,—were he informed that there was a medical department in the army, with numerous surgeons, an efficient head, and an organised system of reporting—were he told that these Reports were avowedly drawn from medical documents and archives in the possession of that same medical department, he would conclude, of course, that some officer in that department superintended, or helped to superintend, the publication of its documents, and that a work essentially medical, was consigned to the care of a professor of medicine. What would be his astonishment at learning that this Reporter General was a mere soldier, conversant no doubt with bayonets, not unacquainted with figures, but possessed of no more medical knowledge than would go to the composition of a black draught!

His astonishment would cease when he learnt the whole truth,—when he was informed that the surgeons of the Queen's service were too often neglected, too commonly set aside, and looked on as necessary yet contemptible appurtenances, like the suttlers or the common women of the camp.

It may be said indeed, that Captain Tulloch has taken to himself the assistance of two medical officers. No doubt he has. He could not do without them. Even a Heaven-born Doctor is obliged to avail himself of the assistance derived from mundane physic. But the editor of these Reports, avowedly and necessarily founded upon medical documents, as necessarily and still avowedly assisted and supported by medical men, takes especial care to place the latter completely in the back-ground, and, instead of being associated with them as equals and co-editors, which would have been as decent as correct, he buries them in a foot-note in the smallest type, while his own name is blazoned in letters so big and in a place so conspicuous, that the very printer's devil must have blushed through all his blackness. Nay, Mr. Marshall and Mr. Balfour are not even complimented by the conventional title of gentlemen, but are styled "the parties," as if they had contracted to furnish Captain Tulloch with the paper or the ink.

### I. THE UNITED KINGDOM.

This part of the Report comprises six sections. For obvious reasons, we shall not attempt to analyse each, but content ourselves with selecting such passages and portions as carry with them interest or instruction.

As the troops of the Line are continually passing to and from foreign service, it is necessary to exclude them as data for the mortality of troops at home. The Reporters consequently confine their observations principally to those regiments of cavalry which have not been serving abroad during the period embraced in this Report, and the Household Troops whose service is for the most part confined to the duties of the metropolis.

It may be loosely said that the diet of each soldier consists of three-quarters of a pound of fresh beef or mutton, which is made into soup with vegetables for dinner, and a pound of bread with coffee for breakfast. Where the regulated stoppage admits of a larger supply, the commanding officer may direct it to be purchased for the soldier if he thinks necessary.

The extent of duty and nature of their employment of course vary according to the arm of the service to which the troops belong, and the circumstances which call for their exertions; but in general it may be remarked that in this country they are seldom employed on any duty not strictly military; fatigue and working parties, which are common in the Colonies, are rarely necessary, and, with the exception perhaps of occasional night duty, there appears nothing in the nature of their occupations likely to operate prejudicially to health. It may safely be affirmed that they are at least better lodged, better fed, and have less onerous duties to perform, than the great mass of the labouring population. It might therefore be supposed that the mortality of the soldier was below that of the civilian, *selected* as the former is, with all the attributes and characters of health.

From all these circumstances it might fairly be supposed that the mortality of these troops would be much lower than that of civilians. But it is not.

By the Regimental Returns of the ages of the Dragoon Guards and Dragoons, it appears that nearly one third, were between 18 and 25, another third between 25 and 33, and the remaining third of various ages between 33 and 40, with the exception of a few boys under 18. It may therefore be safely assumed that the average age of this class of troops is from 29 to 30; now, by the Carlisle tables, which exhibit the mortality of this country in the most favourable light, the number annually deceasing out of a thousand persons of that age would be about 10.

By Mr. Finlaison's observation, deduced from the duration of life among the government annuitants, about 13.

If we take the mean between these, *viz.*, 11·5 per thousand, as our standard, it corresponds very nearly with the ratio deduced from the Population returns, and is sufficiently accurate for the object in view.

The ratio of mortality among the troops in question would amount, according to tables and calculations furnished by Lieutenant Tulloch, to about $13\frac{3}{10}$ per thousand, or from 1830 to 1836, years when the cholera or influenza prevailed, $15\frac{3}{10}$.

Comparing this, observes Lieut. Tulloch, with the rate of mortality ascertained from the preceding table, we should arrive at the conclusion that the proportion of deaths is at least one third higher among these troops than among an equal number of civilians of the same age, though the former have been carefully selected for their robust frames and supposed vigour of constitution.

This would at first sight indicate that the military profession, even under the most favourable circumstances, operates prejudicially to the health and constitution of those employed in it; and we should have proceeded to investigate the causes to which that was attributable, were it not in some measure accounted for by the great difference between the mortality in populous towns where troops are generally quartered and what prevails throughout the whole kingdom, or in the rural districts, on which the previous calculations as to the duration of civil life have principally been founded.

The great disproportion between the mortality of a town and country population has of late years been made the subject of Parliamentary investigation, and various returns illustrative thereof have been ordered from the principal towns in the kingdom, which unquestionably prove the ratio there to be nearly one-third higher at the prime of life than among the rural population.

Our author compares the mortality of the troops with that of the population of Chester, Leeds, Bolton, Bury, Preston, Wigan, Bradford, Stockport, Macclesfield, Glasgow, London, and a few more towns. Omitting the table which contains the data, we may add the Reporter's conclusion that :—while the mortality among the Dragoon Guards and Dragoons, supposing the medium age to be 30, has been $15\frac{3}{10}$ per thousand, that of the civil population in the same towns, even between the ages of 20 and 30, has been 16 per thousand—a sufficient evidence that the apparent high ratio among the troops, as compared with the general mass of the population, arises not so much from any deteriorating influence in their profession, as from the disadvantage of their being subject to the insalubrious atmosphere of densely-populated districts.

Capt. Tulloch adds, that after making every allowance, and taking every circumstance into consideration, it is certainly remarkable that the mortality should be so high among a class of men selected with such care.

He has not deemed it necessary to enter into any investigation as to the relative mortality of the troops in England, Scotland, and Ireland ; but from a rough estimate it appears that the troops in Scotland have been rather more healthy than in England or Ireland.

The Reporter next enters on the consideration of the amount of sickness in the Dragoons. But so many liabilities to error beset us in investigating this particular question, that we do not conceive it would be worth our while to enter on it. It appears that there is no class of diseases, save those of the lungs, from which the troops suffer in a greater degree than the most select of the population.

*Prevalence of Suicide among the Military.*—" The large proportion," says the Reporter, " of suicides among this class of the military is a subject which particularly claims attention. Out of a total of 686 deaths no less than 35, or upwards of 1 in 20 of the whole, have been from this cause alone, independent of many attempts which did not prove fatal ; whereas among those insured in the Equitable the proportion of suicides was not one-fifth part as great, being only 1 in 110 of the deaths. This extreme tendency to self-destruction in the army will best be estimated by a comparison with the proportion of suicides in civil life in different countries, as stated by a recent statistical author.

| | | | | |
|---|---|---|---|---|
| In France there is annually | 1 | suicide to | 18,000 | inhabitants. |
| Prussia ,, | 1 | ,, | 14,404 | ,, |
| Austria ,, | 1 | ,, | 20,900 | ,, |
| Russia ,, | 1 | ,, | 49,182 | ,, |
| State of New York | 1 | ,, | 7,797 | ,, |
| ,, Boston | 1 | ,, | 12,500 | ,, |
| ,, Baltimore | 1 | ,, | 13,656 | ,, |
| ,, Philadelphia | 1 | ,, | 15,875 | ,, |
| Dragoon Guards & Dragoons in the United Kingdom | 1 | ,, | 1,247 | |

In cities, where a large proportion of the military are quartered, the ratio of suicides is greater than in the whole population of a country, but still much below that among our troops. In the Department of the Seine (Paris) for instance, between 1817 and 1825, the suicides averaged annually—

| | | |
|---|---|---|
| | 1 in 2,400 | inhabitants. |
| In Berlin, from 1813 to 1822 | 1 ,, 2,941 | ,, |
| Geneva, ,, 1820 ,, 1826 | 1 ,, 3,900 | ,, |
| London . . . . . . . | 1 ,, 5,000 | ,, |

Even assuming, however, the very highest average in civil life in this country, it would appear that suicides are at least five times as numerous among this class of the military. It is necessary, however, to keep in view, that instances of self-destruction rarely occur among persons under the age of 18, and are by no means so frequent among females as males—circumstances which must materially influence any comparison between its prevalence among a population of all ages and sexes, and a select body of troops from 18 to 40 years of age.

The proportion of suicides is found to be higher among the Dragoon Guards and Dragoons than any other description of force, probably because these corps contain more of that class who have by dissipation or extravagance reduced themselves from a higher sphere in life to the necessity of enlisting, and on whose minds this change of condition may, in some instances, operate so powerfully as to lead to self-destruction."

Making every allowance claimed by the Reporter, the number of suicides among the Dragoons is still lamentably striking. Perhaps reflection on the *morale* of the army may tend to diminish our surprise. In this country, we fear that the virtue of the land is not to be found in the military ranks. *They* frequently enter the army, *who* have proved themselves unfit for the trusts of civil life. The roué, the prodigal, the vain, the reckless, and the idle too commonly hail the recruiting officer. The indolence of the soldier's life begets the listlessness of ennui, and the desperate relief of sensuality; and the fatigues of a lazy occupation are lightened by the recreation of seduction and debauchery. One of our greatest statesmen warned his son, Robert Cecil, against the profession of arms; for soldiers in peace, said he, are like chimneys in Summer.

The sickness, the mortality, and the suicide of the dragoon, may disabuse perhaps the mind of the impression made by a red coat and a glittering sabre. That gay outside conceals and fosters the seeds of moral and of physical disease! The happy soldier—

> Who lives on his pay,
> And spends half-a-crown out of sixpence a-day, when subjected to the severe and exact scrutiny of statistics, is stripped of his tinsel, and becomes an object of serious consideration. The philanthropist naturally asks, whether this state of things must, of necessity, be—whether men selected in the prime of life, and in the vigour of health, well fed, well clothed, taxed with light labour, oppressed with no care, no thought for the morrow's bread—whether such, we say, should necessarily be as sickly, as short lived, as frequent suicides, as these Reports establish that they are. This question is one for those on whom the management of the army depends, to answer to their own and the nation's satisfaction. We believe that it is the *morale* of the soldier that requires amendment; and the first step has been taken in the reformation of that, by establishing booksocieties in the barrack. The school-master must penetrate there, before the *cat*, the drummer, and the "crowner's quest," will depart.

*The Foot-Guards.*—The average strength of these troops may be stated at 4,764. The mortality, the Reporter writes, has been on the average of the seven years, 1830 to 1836, $21\frac{6}{10}$ per thousand of the strength annually, being nearly one-half higher than in the Dragoon Guards and Dragoons. This is the more remarkable, as it has been shown that the climate of London, though certainly much less favourable to health than that of the rural districts, is not more insalubrious than many of the other towns in which the troops are quartered throughout the kingdom; the average annual mortality of the civil population between the ages of 20 and 40 being under 15 per thousand, and that of the East India Company's labourers employed there as low as $12\frac{1}{2}$ per thousand at the same period of life.

The Reporters add, that "notwithstanding all the disadvantages of frequent night duty to which the Metropolitan Police Force is exposed, the mortality out of an average strength of 3400 constantly employed was but 30 per annum, being under 9 per thousand, in addition to which nearly the same proportion was invalided for bad health. As it is understood, however, that many leave that service of their own accord if they find it proving injurious to their constitutions, we forbear drawing any positive conclusions from this source, except that it certainly tends to strengthen the opinion that the great mortality of the Foot Guards is attributable to other causes than the climate of the Metropolis, especially as we shall hereafter have occasion to show that the Household Cavalry do not suffer in a similar degree from its influence."

A Table proves that the great mortality of the Foot Guards arises entirely from diseases of the lungs.

"It would appear that this high ratio of mortality by diseases of the lungs among the Foot Guards is not a necessary consequence of residence in the Metropolis, but rather originates in some peculiarity in the moral or physical condition of that description of troops, from which the others are comparatively exempt; for by calculations deduced from the London Bills of Mortality, from 1830 to 1835, it has been ascertained that out of a thousand deaths among the civil population the number by diseases of the lungs were,—

| | |
|---|---|
| Pleurisy . . . . . . . . . . | 12 |
| Influenza . . . . . . . . . . | 1 |
| Inflammation of Lungs . . . | 96 |
| Consumption . . . . . . . | 177 |
| Asthma, &c. . . . . . . . | 42 |
| Total . . . . | 328 |

being scarcely one-third of the whole; whereas out of 745 deaths among the Foot Guards no less than 487, or upwards of two-thirds, were from these diseases.

But the most conclusive proof on this subject is, that no such peculiarity is manifested in the fatal diseases of another class of troops *also quartered in the Metropolis*,"—namely, the

*Household Cavalry.*—The average mortality of this class of troops is 14·5 per thousand. Thus, though exposed to the climate of the Metropolis, the mortality is not so high by at least one-half as among the Foot Guards, and is even lower, by a small fraction, than among the cavalry corps employed throughout the kingdom. The mortality by diseases of the lungs is but 8·1, while among the Foot Guards it has averaged 14·1 per thousand annually during the same period. That the exposure of the Foot Guards in a greater degree on night duty will scarcely account for this difference may readily be supposed from the fact that, even among the troops of the line serving at home, whose constitutions have in some instances been deteriorated by residence in tropical or unhealthy climates, and who have an equal share of night duty to perform, the mortality by diseases of the lungs is much lower.

The Reporter next passes to the consideration of the liability of the troops to the principal diseases which carry them off.

1. *Fevers.*—Taking the average of the Dragoon Guards and Dragoons, the Foot Guards, the Household Cavalry, and Civil Life, 1·6 per thousand may be computed to die annually from fever.

From an extensive series of observations, it has been ascertained that, among troops serving in this kingdom, fevers are more prevalent during the summer than the winter months, in the proportion of 5 to 4. Of 4499 attacks, of which the dates have been stated, 2531 took place between May and October, and only 1968 during the rest of the year. This is by no means uniformly the case, however, for in 1832 and 1834, the preponderance of febrile cases was in those months which in other years were most exempt from them.

2. *Eruptive Fevers.*—This class of diseases is now of rare occurrence in our army. About 2 out of every ten thousand of the troops throughout the kingdom, die annually from this class of diseases.

The admissions amount to about 3 per thousand annually, and the proportion of deaths to admissions is 1 in 15. It may be necessary to state, that by the regulations for the management of Regimental Hospitals, the Surgeon is called upon to report half-yearly, that every man, woman, and child, belonging to the regiment, bears unequivocal marks of either small or cow-pox, and he is directed to keep a register of the names and appearances, on the days of examination, of all patients vaccinated. Nothing shows the advantage of this precaution better than the fact that, in the Dragoon Guards and Dragoons quartered throughout the kingdom, only one soldier has been attacked by small-pox in every two thousand annually.

3. *Diseases of the Lungs.*—The average mortality throughout our army from this cause may be stated at 8 per thousand annually. The rarity of recovery from consumption, says the Reporter, is strikingly exemplified in the fact that of 286 admissions, among the Dragoon Guards and Dragoons, 236 are ascertained to have proved fatal, and the remaining 50 are more likely to have consisted of re-admissions than recoveries.

Nearly four-fifths of the fatal cases of diseases of the lungs arise from consumption, being as many as from all other causes in the army at home. The highest estimates in civil life rate the mortality from this disease at a seventh part of the deaths of all ages : or if the observation is confined to adults alone, it may possibly amount to a fourth part, being at the utmost only half as high as among the troops.

That soldiers should suffer so much from this disease is remarkable, as an active life, spent for the most part in the open air, has generally a very material tendency to counteract its influence. From some very extensive calculations by Mons. Lombard, on the influence of professions on consumption at Geneva, it has been found that persons whose occupations were of an active description, inducing muscular exercise, and carried on in the open air, were not half so liable to that disease as those whose occupations were sedentary or carried on in shops and manufactories.

If the aggregation of a number of men into one apartment, even though the space is not very confined, creates a tendency to this disease, then it clearly points out the propriety of affording the soldier as ample barrack accommodation as possible, not with a view to his comfort alone, for that is a matter of minor consideration, but in order to check the ravages of a disease which creates more mortality than all the others to which troops in this country are subject.

4. *Diseases of the Liver.*—Very rare. The ratio of admissions has been about 8 per thousand annually, and the proportion of deaths to admissions may be estimated at 1 in 18.

5. *Diseases of the Stomach and Bowels.*—Nor are these a source of great mortality. The average of the whole may be taken at about $\frac{5}{10}$ per thousand or 5 in ten thousand annually ; and it is especially worthy of remark that, though in many of our colonies we find the mortality among our troops by this class of diseases not only higher than in civil life, but in some cases even as high as by all the diseases in civil life together, so far from there being any corresponding feature among the troops in this country, it is even less than among the most select of the population.

6. *Epidemic Cholera.*—During the three years it prevailed, about 2·8 per thousand of the strength were cut off by it annually.

Nothing can be more remarkable than the undeviating regularity with which this disease seems to have exerted its fatal influence in all localities. Here we have instances of different bodies of troops quartered in various situations throughout the kingdom, and yet the proportion of deaths is to within a fraction the same among all.

This is, we should think, a sufficient commentary on the thousand and one successful plans of treatment. The hot water and the cold—the calomel and salts—the stimulants and sedatives—the contagionist and anti-contagionist seem all to have been pretty nigh on a par. The mortality, we should observe, increased progressively with the advance of age.

7. *Diseases of the Brain.*—" We shall assume $\frac{8}{10}$ per thousand, or 8 in ten thousand, for our standard of the annual extent of mortality by this class of diseases among troops in the United Kingdom, being only half the proportion which occurs in civil life, as shown by the Tables of the Equitable Office, on page 8. But this class of diseases is generally supposed to prove a greater source of mortality among the higher ranks, of whom the persons insured in that office are principally composed, than among the lower orders from whom our soldiers are recruited." 14.

8. *Dropsies.*—About $\frac{4}{10}$ per thousand are taken as the average number of deaths from this source.

*Mortality of Different Ages.*—The mortality of the Dragoons increases progressively with the advance of age, but by no means in so rapid a ratio as in the West India stations. But with the *Foot Guards* there will be observed this remarkable difference, that the mortality falls in a much higher proportion on soldiers between 18 and 25, and 25 and 33, in the Foot Guards than in the Dragoon Guards and Dragoons, while between 33 and 40, the mortality in both these descriptions of force is very nearly alike. This may be held in some measure to arise from a larger proportion being annually discharged for disabilities in the Foot Guards than in the Cavalry, whereby the few who are left above 33 must be comparatively more select lives in the former than in the latter force, and consequently less liable to mortality.

But though this may account for the reduced ratio of mortality between 33 and 40, to what are we to attribute its excess between the ages of 18 and 33, at which period of life it is double that of the Dragoon Guards and Dragoons or even of the Household Cavalry quartered in the Metropolis, while the number discharged for disabilities under 14 years' service is also proportionably high? This question is not satisfactorily answered.

*On the Mortality among Officers serving in the United Kingdom.*—The data, on this head, are imperfect, many officers selling out when their health begins to be impaired. But, making allowance for this and for other disturbing circumstances, the Reporters have ascertained from the Army List that, from 1826 to 1836 inclusive, there died among the officers of the Household Troops, Dragoon Guards and Dragoons, serving in the United Kingdom .......................................... 67

The average number of officers in these corps, exclusive of the staff after-mentioned, was about .......................... 700

Making the annual ratio of mortality during these eleven years $9\frac{1}{2}$ per thousand of the strength, or very nearly the same as among the most select class of individuals insured in the Equitable Office between the ages of 20 and 40.

During the same period there were on the average about 27 regiments of the line always on the tour of home service; of the officers of which corps there died.................................. 110

The average number of officers belonging to these corps, exclusive of the staff after-mentioned, was.................................. 900

Making the average ratio of mortality during these eleven years, 11 per thousand of the strength annually.

In this calculation they have excluded the Paymasters, Quarter-Masters, and the Medical Staff, as they are considerably above the average age.

*On the Influence of the Seasons in producing Sickness and Mortality among Troops in the United Kingdom.*—From the absence of sufficiently minute returns of the Household Troops, the Dragoon Guards and Dragoons alone offer data for determining this point. The Reporters present a table of the admissions for and deaths from acute diseases, in every 1000 of those troops during each month of the year. This table shews that, notwithstanding the large proportion of diseases which are attributed to the changeable nature of this climate, the attacks of sickness are fewest among the troops during the fogs and gloom of November, and throughout the winter they are considerably under the average, even though, from their professional duties, soldiers are much exposed to its severity; while during the months of July, August and September, when there exists that mild and serene atmosphere which *à priori* might be supposed most conducive to health, the proportion of sickness generally attains its maximum. The same feature will hereafter be shown to exist to a still greater extent in America, and other regions of the northern temperate zone. April and May seem also to prove peculiarly fatal to chronic cases among the troops in this kingdom ; but at least nine-tenths of these are from consumption, and the influence of spring in accelerating the course of that disease has been often the subject of remark.

On comparing these results with what have been observed in the French army, they are found to tally in a remarkable degree. On inspecting a similar table of the mortality in that army, it appears that, whatever may be the causes which give such an unhealthy character to the autumnal season, these operate no less powerfully in the French army than our own, only that they seem to be a month later of coming into operation, and continue for a month longer than in this country. This, however, may possibly arise from a difference in the periods embraced in the Returns; for instance, if they are made up at the commencement of each month, the deaths there stated will be those of the previous month, and if this distinction has not been attended to in the above calculation, the necessary correction would make the results as to the influence of the seasons correspond in both countries, *viz.*, July, August, and September would be the most sickly, and November nearly the least so.

Mons. Quetelet, a statistical author whose attention has been drawn to this peculiarity of the mortality among troops being so much higher during the autumnal months than at any other period, has recently been at great pains to ascertain whether the same law extends to persons in civil life at corresponding ages. On inspecting his table of the mortality in each month, among persons between 20 and 40 in Belgium, it appears that, at those periods of life corresponding to the average ages of our soldiers, the autumnal months instead of being, as in the army, the most fatal, are, in civil life, the reverse; when, therefore, we find the unhealthy character of that season established in a still more marked degree among Troops in the colonies, we shall no longer be inclined to view it altogether as a peculiarity of climate, but as in some measure resulting from the operation of a general cause affecting the health of soldiers in all latitudes north of the line.

### II. On the Sickness and Mortality among Troops serving in the Mediterranean.

The Mediterranean Stations form three distinct Military Commands—Gibraltar, Malta, and the Ionian Islands.

1. *Gibraltar.*

On the topography of this celebrated Rock we need not dwell. We may simply observe that the climate of Gibraltar, though dry and sultry in summer, and subject to fogs and mists throughout the year, may generally be characterised as healthy. The greatest height of the thermometer in the shade during a period of five years was 91°, and the minimum 50°. The temperature in summer is always from 3° to 4° lower during the night than during the day—often much more; and in the morning before the sun appears above the Rock, and also towards sunset, the air is pleasantly cool and refreshing, even in the hot season.

Though snow seldom or never falls, and ice is rarely formed, the cold is keenly felt during the winter months, especially by those who have been long resident on the Rock.

The prevailing winds are from the westward and eastward. It seldom blows from the north or south, and when from these quarters continues but for a short time. The westerly winds are clear, dry and refreshing; blowing directly on the town, they promote a free circulation of air, and are esteemed highly favourable to health. The easterly winds, or Levanters, as they are termed, have quite a contrary character; their baneful effects are said materially to aggravate wounds and acute diseases, and often to prove fatal to convalescents; being surcharged with moisture during their transit across the Mediterranean, they are always damp, raw and unpleasant, and when from the southward of east, are generally accompanied with thick fogs, which envelop the Rock, and are supposed to produce the same debilitating effects as the sirocco in the upper part of the Mediterranean.

Easterly winds are most prevalent from July to November; this is the unhealthy period of the year among troops in Gibraltar, but as the same is the case in other countries remote from the influence of these winds, the insalubrity of that season cannot be altogether attributable to their agency.

The rains generally commence in the end of September or beginning of October, and set in with such violence as frequently to overflow the water-courses and commit great havoc in the streets of the town. The succeeding rains which continue to fall at intervals till the end of May, are much lighter, and during the middle of summer there is seldom any, the sky is then without a cloud, vegetation becomes languid, and, unless irrigated by artificial means, generally perishes.

Except during the years 1817, 1818, and part of 1819, when the 4th West India Regiment (a black corps) was employed at this station, the garrison has generally consisted of the service companies of five regiments of the line, with five companies of Artillery and one of Sappers and Miners.

Owing to the great extent of the works, and the number of sentries required, the duties of this garrison are rather severe: the results in this Report, however, sufficiently establish that they are by no means so much so as to prove injurious to health.

The mean strength of the white troops from 1818 to 1836 has been 3,172. The average ratio of deaths amounts to 22 per thousand of the strength annually, being considerably above that of troops in Britain; but in this is included the mortality occasioned by two severe and fatal epidemics—one of yellow fever in 1828, and another of cholera in 1834, by which nearly as many deaths occurred as during all the other 17 years at this station. Exclusive of these, the mortality would not have been more than 13 per thousand annually.

Sufficient evidence of the general salubrity of Gibraltar is afforded by the low mortality among the civil inhabitants. Out of a population of from 16,000 to 17,000, the deaths recorded in 10 years, when no severe epidemic prevailed, did not average more than 350 annually, which is as low as in the United Kingdom, though the fluctuations constantly occurring in the number of the inhabitants prevent any calculation as to the exact ratio. The principal diseases would seem to be fevers—diseases of the lungs—diseases of the stomach and bowels—abscesses and ulcers—and diseases of the eyes.

Passing to particular diseases we naturally are arrested by the :

*Yellow Fever.*—Disregarding the vexata quæstio of its importation or spontaneous origin, we perceive and quote the following facts on its several appearances and its mortality—facts alike interesting and alike important to all parties :—

"The first appearance of this epidemic, of which we possess any specific details, was in August 1804, but till the end of September it did not become so prevalent as to call for the notice of the public authorities. It then rapidly increased till the end of October, when it reached the maximum, after which it gradually diminished in frequency and severity, and ultimately disappeared in the end of December or beginning of January. In this instance, however, it seems rather to have died away from want of subjects than from any mitigation of the causes by which it was induced,—for, after the most diligent inquiry, only 28 adults could be discovered within the garrison who had escaped the malady, and it very rarely attacks the same person twice. A manuscript journal of the events of that period states that neither wind, rain, nor any change of weather, had the smallest effect in checking its ravages or diminishing its malignity ; about one third of the troops who were attacked died, and of the civilians a still greater proportion. The total number of deaths during its continuance amounted to—

| | |
|---|---|
| Officers .. .. .. .. .. .. .. | 54 |
| Soldiers .. .. .. .. .. .. .. | 864 |
| Soldiers' wives and children .. .. | 164 |
| Civilians .. .. .. .. .. .. .. | 4864 |

We cannot state the precise number of each class so as to ascertain the ratio of mortality, but about a fourth part of the troops and more than a half of the civil inhabitants were cut off. To the precaution of encamping the former out of town, which was adopted on the 26th of September, is probably to be attributed their not having suffered to so great a degree as the civilians who remained constantly within the garrison.

In the end of October 1810 a similar disease appeared, but was confined to the soldiers of one regiment, of whom only six died, and it ceased on that corps being encamped on the Neutral Ground.

In 1813 this disease again made its appearance as early as the middle of July, but two months elapsed before it prevailed to such an extent as to form the subject of official Reports. In the month of October most of the troops were removed to encampments on the Neutral Ground, where they were in a great measure exempt, though it continued to rage in the town till the month of December. It then became extinct, after cutting off 461 of the troops and 883 of the inhabitants.

In the following year this epidemic again broke out in the month of August, and, the same precaution of encamping the troops having been resorted to, it disappeared by the end of October, with the loss of 114 of the military and 132 of the inhabitants.

The garrison suffered from no similar visitation from this period till 1828, when that epidemic broke out which is referred to in the preceding Tables.

The first cases occurred among the civil inhabitants in the southern district of the town about the end of August, and on the 5th of September its appearance among the Sappers and Miners and 12th Foot quartered in that neighbourhood was officially reported: the men of the latter corps were immediatly encamped on the Neutral Ground, from which period till the end of the month, when they were obliged to resume their duties in the town, no new cases occurred among them.

Notwithstanding this precaution, however, by the middle of September the disease had appeared throughout all the town, and though both on this and previous occasions its ravages were at first confined to the filthiest and most densely populated districts, yet ultimately all ranks suffered from it in nearly an equal degree."

"The proportion of deaths to the number treated was almost the same among the inhabitants as the troops, but the proportion attacked does not appear to have been so great. This exemption is, however, more apparent than real, as from the total population should be deducted 6,000, who, having had the disease formerly, may have been thereby exempt from it on this occasion,—with this correction, its relative prevalence among military and civilians was very nearly the same."

The Reporters observe that, in 1828 and also in 1813 the mortality attained its maximum on the 16th of October, and in 1804 about a week earlier. The same has been observed in other parts of Spain, and also at New York, when yellow fever prevailed.

There was one marked peculiarity about it to which they consider it necessary to advert. In Gibraltar the same individual has seldom been attacked twice, even though a long series of years may have elapsed since he first suffered from it; but in the West Indies, and on the West Coast of Africa, a former attack of remittent fever secures no such immunity,—indeed it could be proved, by reference to the Returns from these stations, that, in many corps every soldier must have been treated, on the average, twice or thrice for remittent fever during the few years of his service there.

There was nothing remarkable in the atmospherical phenomena, which preceded the last epidemic. The Reporters go into this part of the subject and prove, we think conclusively, that to no appreciable atmospheric agency could the epidemic be attributed. Though no marked diminution either in prevalence or severity could be distinctly traced to the occasional reductions of temperature which took place during the continuance of this disease, yet it may be proper to observe that these epidemics have never been known to make their appearance at this station during winter, and have always declined in severity as that season approached.

The Reporters conclude with some observations upon the cause of the epidemic. If they leave it in great uncertainty, they do no more than might have been expected. More positive opinions would have been contradicted by the very facts they deal in.

"As a cause of this disease, much has been attributed to the want of due ventilation in Gibraltar during the hot season, owing to the town being so much screened by the rock in rear; but this cause ought obviously to operate equally in all years under equal degrees of temperature, which it does not; and it is only necessary, as a complete refutation of this theory, to refer to the situation of Cadiz and many of the other towns along the coast of Spain, which, though open to every breeze, are more frequently subject to this disease, and that too in a more aggravated form, than Gibraltar.

In 1804 and 1813 the state of the drains, and the crowded and filthy condition of the town, were assigned as the primary causes of the epidemic; but in 1828 many improvements had been made in these respects, and though some complaints were urged against the drains, and one was undergoing repair during the Summer, it does not appear that, at the time the disease broke out, they were in a worse condition than they had often been in previous years when the town enjoyed its usual salubrity.

A removal, even to a very small distance from the town, seems to have secured a complete immunity from the epidemic, for it is stated by the Surgeon of the 12th Regiment that of 92 women and 190 children encamped on the Neutral Ground during the whole period it prevailed, not one was attacked, though in constant communication with the soldiers of the corps who went daily from that encampment to do duty in the town, and of whom many were attacked with the disease on their return. It is also remarked, that on the Neutral Ground, the epidemic never made its appearance to any extent, though from 6000 to 8000 of the inhabitants were encamped there, yet that spot is composed of materials which are supposed highly favourable to the formation of malaria.

By those who maintain the doctrine of contagion, the disease was supposed to have been imported from the Havannah in a Sweedish ship, which lost two of her men on the passage, but on investigation, it was not established that the disease of which they died was yellow fever; and whether the origin of the epidemic be attributable to contagion or not, there seems no good reason for assigning the introduction of it to that ship more than to many others, especially as, on the suspicion of having had yellow fever on board, it underwent six weeks' quarantine, and was twice fumigated before being allowed to hold any communication with the garrison; during all this period no deaths or sickness took place on board, and at least ten weeks elapsed between the death of the two sailors and the period when the epidemic became prevalent.

By those adverse to the doctrine of contagion it has been asserted, that persons in immediate attendance on the sick within the garrison did not suffer to a greater extent than others who were not so exposed, and that those employed on that duty beyond the walls were not at all affected by the epidemic. Having no return of the number thus employed, or of the casualties among them, under these circumstances, it is impossible to test this assertion by the numerical evidence requisite to warrant its authenticity.

Such are the pricipal facts connected with the appearance and progress of this singular disease, of which the cause still seems involved in mystery. When the unhealthy character of the autumnal months throughout the globe has been distinctly traced in the course of these investigations, and the remarkable coincidence established that most of the epidemics of a fatal nature in the northern hemisphere have either made their appearance, or raged with the greatest severity, about that season of the year, it may possibly lead to the discovery of some peculiarity in the constitution of the atmosphere at that period, which may tend to elucidate the subject."

*Diseases of the Lungs.*—The ratio of admissions, say the Reporters, for this class of diseases is to that in the United Kingdom as 141 to 148, the principal difference being, that catarrhal affections are less frequent in Gibraltar, while inflammation of the lungs is much more so; the cases of the latter are, however, of a milder character, as only 1 in 45 died of those admitted into hospital in Gibraltar, while 1 in 18 died of those admitted for the same cause among the Dragoon Guards and Dragoons in the United Kingdom. The total mortality by diseases of the lungs would appear to be less at this station than at home; but that, we apprehend, arises from many of the consumptive patients being invalided, who if they die on their passage, or after their arrival in Britain, are not included in the Returns of the station where their diseases originated. That this is sufficient to account for the difference may easily be supposed from the fact stated in the Medical Report of 1835, that during the thirteen years previous, the average number of deaths from consumption in Gibraltar was $12\frac{3}{10}$ annually, besides about five sent home labouring under the same disease, of whom few or none recovered.

Consumption is as prevalent and fatal among the civil as the military population.

The influenza, of 1833, made its appearance in this garrison about the middle of December. But only two died of it. By the 8th of January the admissions had materially decreased, and by the 14th had entirely ceased. During its continuance, as well as for some time before it broke out, there was a prevalence of dry winds from the N.E. and N.W., and the medium range of the thermometer during the day was from 60° to 62°, but the nights were very cold. The disease entirely disappeared on the setting-in of the first rains, accompanied by easterly winds.

*Diseases of the Liver.*—This class of diseases is by no means either prevalent or a source of great mortality in Gibraltar, indeed it is only in a very trifling degree more so than in the United Kingdom.

*Diseases of the Stomach and Bowels.*—Comparing the influence of these diseases among the troops at this station and in the United Kingdom, it would appear that they are twice as prevalent and about thrice as fatal. This is attributed by the Reporters to the quantity of salt meat issued to the troops.

*Epidemic Cholera.*—Admitted, 459—Died, 131—Proportion of Deaths to Admissions, 1 in $3\frac{1}{2}$.

We shall content ourselves with mentioning one or two circumstances which bear upon the question of contagion.

During the Spring of 1834 this disease prevailed in several of the towns and villages in the vicinity of Gibraltar; but no case occurred in the garrison till the 23d of May, when a soldier of the 92d Regiment, quartered in the King's bastion barrack, was suddenly attacked with decided symptoms, from which, however, he ultimately recovered. Two of the civil inhabitants died from it on the 3d and 5th of June, several other cases occurred on the 14th, and by the 17th of that month its presence was so decidedly manifested, both among the troops and inhabitants, as to be made the subject of official reports. From that period the number of cases progressively increased till the middle of July, when they began to decline, and the disease became extinct in its epidemic form in the beginning of August, though bowel-complaints of a severe character prevailed among the troops for several weeks after.

The Regiment which suffered most was the 5th Foot, though quartered in the south barracks occupying one of the healthiest situations on the rock, and, by one of those peculiarities which often characterises the progress of this singular disease, its ravages were principally confined to the inmates of the two wings of the building, while those in the centre were comparatively free from it. It was also remarked that though the first case was in the 92nd Foot, quartered in the centre of the town, in immediate contact with the inhabitants, no other occurred in that regiment till the middle of July, when the disease was on the decline, and even then few were attacked compared with the rest of the garrison.

Although, among the civilians, the old, the infirm, and the dissipated suffered, or were said to suffer, most, this was not the case with the troops. No part of the territory was exempt, and change of site, or of air, was of no avail.

"The medical officers seem to have been almost unanimous in their opinion that the disease was not contagious. In the same ward with the cholera patients in the civil hospital were several persons labouring under other diseases, who, although in constant communication with, and frequently in attendance on, those suffering under the epidemic, were in no instance affected by it. In the military hospital, too, it was observed that the orderlies employed in attendance on the sick were not attacked in a greater proportion than others who were not so employed." 14.

"It may also be stated, as another evidence on this subject, that, of 30 medical officers in constant attendance on the sick during the prevalence of the epidemic, all of whom, from the nature of their duties, were subject to great fatigue and anxiety, only one or two exhibited any symptoms of the disease, and then cases were comparatively slight." 15.

Passing over diseases of the brain, dropsies, and some other affections, we pause for an instant at the following statement:—

"In this comparison the rarity of *venereal affections* at Gibraltar is particularly worthy of notice, the proportion of admissions from that cause being scarcely one-third as high as among troops at home, which has a material influence in diminishing the extent of sickness. A reference to the General Abstract, No. I. of Appendix, will show that by far the greater proportion of these diseases are of that description which may not have originated in impure contact, and that in some years, particularly prior to 1824, cases decidedly of venereal origin were almost extinct in the garrison.

This rarity of venereal affections corresponds with what has been observed in the West Indies; but it cannot hence be inferred with the same certainty that the climate is decidedly unfavourable to their existence or propagation, because at Gibraltar, the strictness of the police regulations for the exclusion of all females likely to communicate venereal, may in some measure account for the comparative exemption enjoyed by the troops." 16.

The latter supposition we think the more probable.

### 2. *Malta.*

As to this Island, invalids, illustrious perhaps, are now dispatched, it may be useful to learn a few facts respecting it.

Malta, say the Reporters, being much exposed to the influence of the hot winds which sweep over the deserts of Africa, and the sandy coast of Egypt and Syria, is subject to a higher temperature, particularly during the summer months, than is usual in the latitude of that island; indeed the heat at that period is little inferior to what is experienced in tropical regions. This high degree of temperature exists not only during the day, but, owing to the radiation of the heat absorbed by the rocky surface of the ground, and the thick stone walls of the buildings, continues, with very little abatement, even after the solar influence has ceased; and sometimes, for a period of several weeks together, the thermometer maintains, during the night, the same height as throughout the day, creating thereby a feeling of extreme lassitude and oppression among all classes of residents.

In September there are frequent showers, increasing in frequency during October and November; but from December to February the rain falls with nearly the same violence as in the tropics, and the atmosphere continues surcharged with moisture till March; it then begins to clear, and during the five following months scarcely a drop falls, and the sky is generally without a cloud.

The most prevalent winds in Malta are from the south-east, south, and north-west. That from the south-east, termed the sirocco, is the most common; and the disagreeable effect it produces on the human frame is frequently adverted to in the medical reports. It prevails principally during the autumnal months. There is no regular land and sea breeze, which, in some southern stations, serves materially to modify the temperature.

The island has been generally esteemed very salubrious. But a reference to the mortality tables does not seem to bear out the supposition. For, if we assume (which there are grounds for doing) 100,000 to have been the average during the period referred to in this Table, the annual ratio of mortality would be about 1 in 39, or nearly $2\frac{5}{10}$ per cent., for all ages, while in this country it has been, on the average of the same years, only 1 in $47\frac{1}{2}$, or $2\frac{2}{10}$ per cent.; so that, even as regards the indigenous inhabitants of both countries, Malta is by no means so healthy as Britain, but seems only to enjoy the average salubrity of the states in the South of Europe, where the mortality varies from 1 in 35 to 1 in 40 of the population annually.

But besides the mortality of ordinary years, on which the above estimate is founded, Malta, in 1813, suffered from the ravages of the plague, which cut off 4486 of the inhabitants, between April and November; and in 1837 cholera broke out, and also cut off several thousands. Here, therefore, as at Gibraltar and other stations liable to occasional visitations of pestilence, it is exceedingly difficult to fix exactly the ratio of mortality among the inhabitants, as so much depends on the series of years over which the observations extend.

For several years past, the force stationed in this island has consisted of the service companies of four infantry regiments of the line, two companies of artillery, and the Royal Malta Fencibles, a colonial corps composed of natives.

From tables it appears, that among every thousand soldiers serving in Malta, 1142 cases of sickness have been admitted into hospital annually on the average of the last 20 years, being more by 176 per thousand than in Gibraltar, though the mortality is less than at that station.

"The casualties increase the average ratio of mortality to 18 $\frac{7}{10}$ per thousand of the strength annually, a sufficient proof that the climate of Malta by no means deserves that salubrious character which has, by some authors, been assigned to it. We have already shown, by reference to the mortality among the natives, that it is much the same, in this respect, as the other states in the South of Europe, and the extent of mortality among the troops, during the above period, sufficiently corroborates that deduction. Were it not for the occurrence of the epidemic fever in Gibraltar in 1828, the average mortality there would have been lower than at this station by at least 5 per thousand annually; and we have shown that Malta is by no means exempt from visitations of pestilence in a much more aggravated form, though no instance of it has occurred during the years over which this Report extends."

It is a singular thing that though, as has been already stated, there has been a larger proportion of admissions among the troops than in Gibraltar, yet on investigating the diseases by which they were occasioned we find that the excess is principally among those which seldom prove fatal, such as venereal affections, ulcers, and diseases of the eyes. This accounts for the extent of sickness being greater, though the mortality has been less than at that station.

*Fevers.*—The troops at Malta have suffered more from fevers than those at Gibraltar, and twice as much as those at home.

*Diseases of the Lungs.*—After furnishing a tabular view of their frequency, the Reporters observe:—

"The climate of this island appears from the preceding results to be by no means favourable to persons predisposed to these diseases: the mortality is higher than in Gibraltar, and there is every reason to believe that could we have taken into account the number invalided, and who died on their passage, it would have proved even higher than at home. It is somewhat remarkable that, in a climate where the thermometer never sinks to the freezing point, where the temperature at night is generally within a few degrees the same as during the day, and where those sudden transitions from heat to cold, to which this class of disease is generally attributed in other countries, are exceedingly rare, the ratio of admissions should be only about one-fifth less than in the United Kingdom.

It may serve as a striking illustration how little influence the climate of Malta is likely to have in diminishing the tendency to consumption, that the proportion attacked by that disease among the troops there during the last seven years has averaged $6\frac{7}{10}$ per thousand of the strength annually, while in the United Kingdom, during the same period, the proportion attacked of the Dragoon Guards and Dragoons was but $6\frac{4}{10}$ per thousand annually. Nor is the fatal influence of diseases of the lungs confined to the troops alone; it extends in a corresponding degree to the inhabitants."

Here follows a table, and *it* is succeeded by these remarks:—

"This total of 6664 deaths in 13 years shows the mortality to have been 513 anually, which upon an average population of 100,000 of all ages is about $5\frac{1}{8}$ per thousand of the strength, being scarcely one per thousand less than among the troops, notwithstanding the night exposure of the latter in the course of their military duties.

Though the climate of this island has been supposed favourable to diseases of the lungs, its inhabitants appear to suffer from them nearly as much as those of high northern latitudes; for the Returns of Sweden show that there were only 14,087 deaths from this class of diseases out of the whole population in one year, being in the ratio of $5\frac{6}{10}$ per thousand, or within a fraction the same as in Malta."

Surely this should give some physicians pause. It but adds another page to the chapter on the transportation of consumptive patients to hot climates —transportation to their grave.

*Diseases of the Stomach and Bowels.*—"This class of diseases prevails to a considerable extent: fortunately, however, not in any very aggravated forms: acute and chronic dysentery, which prove so fatal in the West Indies, being here comparatively rare. The annual mortality amounts altogether to $3\frac{6}{10}$ per thousand of the strength, which is considerably higher than in Gibraltar, though the proportion attacked is less. In the Report on the West Indies it was stated that this class of diseases produced comparatively little mortality either among the officers or civil inhabitants; and we were hence led to infer that among the soldiers it was, perhaps, not so much attributable to the influence of climate as to the quality of the diet to which they were restricted. We now possess an indirect proof of the accuracy of these deductions in the fact, that in this island, where the troops enjoy the advantage of fresh provisions, the mortality among them by diseases of the bowels does not exceed that of the civil inhabitants."

The bowel complaints chiefly prevail among the troops from June to November, and nearly the same thing obtains with the civil inhabitants. The Reporters conclude :—

"These documents appear conclusive that diseases of the bowels are intimately connected with, if not induced by, increased temperature, a fact which, though often asserted, has hitherto been without any proof on so extensive a scale: consequently, in all tropical climates in which troops are employed, it is more requisite that their food should be nutritive and easy of digestion, than in temperate or northern regions, as the same quantity of salt meat which might there be consumed with comparative impunity may, from this cause, in warm latitudes, prove an active source of disease. We are well aware that no diet, however simple or well regulated, will secure to the soldier an entire immunity from diseases of this class when exposed to a high temperature in tropical climates; but it is hoped much may be done, by a due attention in this respect, to reduce their prevalence and diminish their fatal influence."

In several of the West India islands bowel complaints have been attributed to the exceedingly moist character of the climate; yet, from June to September, when scarcely a drop of rain falls in Malta, and the sky is without a cloud, the admissions and deaths from these diseases are at their maximum, whereas from December to March, when most rain falls, they are nearly at the minimum.

*Diseases of the Brain.*—In Malta, at all events, the mortality by diseases of the brain is not increased by a high temperature. But it seems at first singular that from June to September, the hottest months in the year, the deaths from apoplexy are scarcely half as numerous as from December to March, which are the coldest; and if the mortality for every year is examined separately, it will be found to exhibit the same feature with remarkable uniformity. The deaths by this disease, too, increase with the utmost regularity from August, when they are at the minimum, to January, when they attain the maximum, and diminish exactly in the same proportion during the other six months of the year. The infantile diseases reported under the head of convulsions follow the same law, though not so uniformly or in so marked a degree.

*Prevalence of Venereal Affections.*—The Reporters state that it is one of the most striking features in the above comparison that the proportion of venereal affections is to within a fraction the same as in the United Kingdom, and more than thrice as high as in Gibraltar. Though there are certainly not the same facilities as in that garrison for preventing intercourse with those from whom the troops are likely to contract infection, yet as the police regulations for the seclusion of all females labouring under venereal are very rigidly enforced in Malta, the difference in the health of the troops in this respect is very remarkable. This peculiarity, taken in connexion with what has already been observed in regard to the rarity of this disease in the West Indies, where no sanatory regulations exist on the subject, confirms the supposition that certain climates may possess an influence in favouring or retarding its propagation, though on what agency that influence depends it is impossible to determine.

We think that this, as well as the prevalence of syphilis upon the Continent, sufficiently prove the inefficiency of police regulations and surveillance. If that inefficiency is apparent in a small colony, how much more obvious must it become in a capital like this. Yet the wish has been privately and publicly expressed, to extend the surveillance of the police to the public women of London. Politically objectionable, if not impossible, it would probably, were it carried into effect, be medically useless.

We may quote one observation of the Reporters, because, mutatis mutandis, what is said of the diet of the Maltese Fencibles and British troops, applies more or less to the European resident in all hot climates, and even to the European in the hotter seasons of his own.

"The Maltese use very little animal food; bread, with the vegetables of the country, and occasionally a little fish, forms their principal sustenance; and the healthy and efficient state of the corps may no doubt partly be attributed to the important circumstance that, in becoming soldiers, they have not been required to change the simple diet which nature seems to have pointed out to the inhabitants of all southern latitudes as most conducive to their health. The cost of the ration to Government is exactly the same as the stoppage from the soldier, so that while the public sustains no loss, the Maltese, even upon his reduced rate of pay, has the same surplus as the British soldier whose habits require a more expensive diet." 29.

*Ionian Islands.*

The islands comprised in this military Command are Corfu, Paxo, Santa Maura, Cephalonia, Ithaca, Zante, and Cerigo. With the exception of the last, which is considerably detached from the others, they extend nearly in a continuous chain from north-west to south-east, skirting the shores of Greece from the entrance of the Adriatic to the southern extremity of the Morea.

The troops employed in this Command consist of the service companies of several regiments of the line, two companies of artillery, and a party of sappers and miners; the force has varied from about 3000 to 4500, according to circumstances.

The climate of these islands has proved much more inimical to our troops than that of the other Mediterranean stations. Compared with Malta the deaths are as $28\frac{3}{10}$ to $18\frac{1}{4}$, and with Gibraltar as $28\frac{3}{10}$ to $22\frac{1}{4}$. Among the natives of the Ionian Islands, the mortality does not appear to be higher than among those of Malta or the South of Europe. Within the last six years, however, a very great reduction has taken place in the mortality of the troops, a circumstance partly, at all events, owing to improved barrack accommodation, drainage, diminution of work for the men, &c.

The prevalence and fatal character of fevers is one of the leading features of the climate of the Ionian Isles—nearly one-half of the admissions and deaths being attributable to that alone. Remittent fever has cut off about 9 per thousand of the strength annually in the Ionian Islands, though not more than 1 in three thousand have died from the same cause in Malta, or even in Gibraltar, if we exclude the epidemic of 1828. Though not so prevalent, this disease is nearly as intense as in the West Indies, 1 in 11 having died of all those attacked, and occasionally at some of the stations even as many as 1 in 3.

The prevalence of remittent fever is in a great measure confined to the months of July, August, September, and October—it sometimes commences in June and continues till November, but rarely occurs during any other period of the year. It is stated that those who have had remittent fever in the summer are particularly liable to intermittent in winter, even if they have removed to a station where the latter is otherwise rare: for instance, after the removal of the 7th Fusiliers to Malta, in 1828, almost every individual who had suffered from remittent in the Ionian Islands was attacked by intermittent; the same was observed in the cases of fever which proved so fatal to our troops at Walcheren.

*Diseases of the Lungs.*—The Reporters make a not unimportant statement, *that*, notwithstanding the variable character of the climate, the rapid alternations of temperature, and the tempestuous weather which frequently prevails in this Command, diseases of the lungs are both less prevalent and less fatal than at Malta or Gibraltar: the admissions into hospital by that class of diseases in these three Commands being respectively as 90, 120, and 141, and the deaths as 4·8, 6·0, and 5·3 per thousand of the strength annually. The principal exemption in the Ionian Islands is from catarrhal affections, which are not half so prevalent or half so productive of mortality as in the other Mediterranean stations, or in the United Kingdom. Most of the deaths arise from consumption; but neither is the proportion attacked so high nor the fatal cases so numerous as in Malta, where there exists a comparatively equable temperature, and that mild condition of the atmosphere which is supposed favourable to persons predisposed to that disease. In Malta, on the average of 20 years, about 6 per thousand of the troops have been attacked annually by consumption, and in Gibraltar and the United Kingdom nearly the same ratio, while in the Ionian Islands only 5 per thousand have been attacked, and the deaths have been fewer in the same proportion. This, however, may be in some degree attributable to the fact, that, in Malta and Gibraltar, one third of the troops are under 25, whereas, in the Ionian Islands, about a fifth only are under that age. Inflammation of the lungs is not more frequent in the islands, where the atmospherical vicissitudes are frequent and sudden, than in Malta.

*Delirium Tremens* is five times as prevalent here as in Gibraltar or Malta. The facilities for indulgence in ardent liquors are greater.

"Here, as in most of the other climates in which there is great facility in obtaining the means of intoxication, the consequences of this vice, as shown in the number of admissions and deaths from *delirium tremens,* are annually becoming more apparent. On an average of the eight years immediately subsequent to 1821, when this disease began to be specially noticed in the Medical Returns, the proportion of admissions and deaths was scarcely one-third as high as during the last eight years included in this Report.

In this fact we have a strong proof that intemperance cannot well be assigned as the principal source of those sudden accessions of sickness and mortality which occasionally take place in these islands, because though it appears to have been materially on the increase in this Command during the last few years, the troops, instead of being more unhealthy, have been rather less so than before. We state this not as an apology for, or encouragement to, intemperance, but to prevent causes being assigned for disease which are not warranted by numerical results." 37.

*Venereal Affections.*—From these there is a comparative exemption in this Command. They are nearly as rare as in Gibraltar, and thrice as much so as in Malta: but this may, in some measure, be attributable to the precaution, of subjecting all public prostitutes to a weekly inspection, and sending those found diseased to a lock hospital. This would seem to contradict an observation which was made in reference to venereal affections in Malta. Perhaps, the limited size of the Ionian Islands may render the business of inspection less difficult than in places stocked with a great population. Perhaps other and less obvious circumstances operate.

*The Plague.*—About the middle of December, 1815, the plague broke out in a small village called Marathea, on the south side of the island of Corfu, a low situation abounding in stagnant pools and marshes, and at all times unhealthy. In the preceding autumn remittent fever had been very common throughout the whole of that district, and the weather, for some months previous to the appearance of the plague, had not been so cold or rainy as usual.

So rapid was the progress of this pestilence, that more than a fourth part of the inhabitants of Marathea were cut off by it in a few days; and before any steps could be taken to arrest its progress, it had begun to show itself in several of the adjacent villages. As soon as its existence was officially reported, measures were adopted for cutting off all communication between the infected districts and the capital; strong cordons of troops and police were posted round each village in which the disease had made its appearance; the inhabitants were shut up in their respective residences; no communication was permitted between them; and the approach to the capital was guarded by a double line of troops, through which no one was allowed to pass from the interior without performing 14 days' quarantine. Owing, it is supposed, to these precautions, the disease was confined principally to the upper and lower districts of Leftimo, in which it originally broke out, and the capital entirely escaped its ravages. It finally disappeared about the middle of May, 1816.

These facts would tell, of course, very strongly, in favour of the doctrine of contagion. They would naturally be trusted and confidently appealed to by the believer in it. The advocate of the influence of unhealthy localities would find some countenance for *his* opinions, in the stagnant pools, the marshes, the insalubrity of Marathea. Each would appeal, the contagionist most confidently, to this particular case, and candid men would admit the appeal to be reasonable.

But let us read on.

"After the plague had ceased in Corfu, it broke out, in the beginning of June, 1816, in the village of Comitato in Cephalonia, in a situation mountainous, rocky, barren, and almost destitute of water and vegetation, the very reverse of the district in which it first made its appearance at Corfu. In a few days it had extended to several families in the village, and so rapidly fatal did it prove, that death followed in every case within a few hours; indeed, in some instances, whole families were cut off in the course of one night. Owing to its appearance not being immediately reported, the same measures which had been resorted to at Corfu for arresting its progress could not be adopted till the end of June; but in the meantime, it did not extend beyond the village in which it first broke out, and entirely disappeared by the middle of July, after destroying from 65 to 70 out of a population of 700. In this instance, the military suffered less than at Corfu, only one case occurred among them, and that terminated fatally."

Nay, even the influence of season proved dubious, or, we should rather say, inert. For the Reporters assert that, unlike the ordinary course of febrile epidemics, which generally become more mild in their character, as they are about to disappear, the last cases of this disease, both at Corfu and Cephalonia, were equally severe and fatal as the first. Nor did the period of its commencement at Corfu correspond with that in which epidemics generally prevail in the Mediterranean, viz. from July to October; on the contrary, it appeared during what is generally the healthiest season of the year. In neither case did the weather exert the slightest influence on the character or progress of the disease: it was just as virulent in the middle of winter in Corfu as in the middle of summer in Cephalonia.

The Reporters next examine in detail, the medical statistics of Corfu, Paxo, Santa Maura, Cephalonia, Ithaca, Zante, Cerigo, and Parga. Into these we do not deem it requisite to go, and we turn accordingly to the General Summary, which presents us with an abridged statement of the main results. From this summary we glean, as the principal fact, that instead of extending over the whole Command as they would be likely to do if resulting from the operation of any general cause, the sudden accessions of mortality which occasionally take place are frequently confined to one island, while others, in the immediate vicinity, are exempt. In 1818, for instance, when 50 per thousand of the strength died at Zante, the mortality at Cephalonia, only a few miles distant, was but 9 per thousand. In 1817 and 1819 the troops were unhealthy both at Santa Maura and Zante, while those of Cephalonia, lying between them, experienced no more than the usual degree of mortality, and in 1828 and 1829, though a fourth part of the force was cut off at Santa Maura, those at Corfu and Zante were subject to no great degree of sickness, and even those in Cephalonia, immediately adjacent, did not suffer in any marked degree. Various other instances of a similar nature might be quoted.

### *Deductions from the Preceding Report.*

The Reporters observe, that the mild climate of the Mediterranean has generally been considered favourable to the cure or prevention of consumption and other pulmonary affections. To ascertain whether this supposition is well founded, or the reverse, is manifestly an object of much importance to medical science, and can only be determined by investigations extending over a long series of years, and including large masses of individuals. The experience of civil practitioners, however carefully recorded, is on too limited a scale to warrant general conclusions on a subject of such magnitude; yet, hitherto, no other source of information has been available for that purpose, and it is not surprising, therefore, if their conclusions, when submitted to the test of numerical calculation, are, in many instances, found to be erroneous.

They go on to remark:—

"We have already alluded to this subject in a more general way, in the course of our observations on diseases of the lungs in each of the Mediterranean Commands, but we can now speak with more certainty from results extending in each instance over the same seven years, and embracing a large number of individuals of the same profession, the same age, the same habits, and having, except at Gibraltar, the same diet. This affords so accurate a standard of comparison as to place beyond a doubt the interesting fact, that, except in the Ionian Islands, the liability of troops to consumption in the Mediterranean stations is even greater than in the United Kingdom. We have not compared the deaths by consumption for a similar period, because conclusions could not have been drawn in regard to the relative mortality with the same accuracy, so many labouring under that disease having died on their passage home or after their arrival in this country; but from all the information we have been able to obtain there can be no doubt that if due allowance is made for these casualties, the proportion of deaths also, among those attacked by consumption, will be found fully as high in the Mediterranean as in the United Kingdom.

We might have carried this comparison further, and shown how little influence temperature has on this disease by the fact that it is still more prevalent and fatal in the mediterranean than in North America, where the soldier has frequently in the course of his duty to be exposed to the night air, when the thermometer is several degrees below zero."

These facts, they continue, offer a striking contradiction to the popular idea regarding the influence of sudden atmospherical vicissitudes, and rapid alternations of temperature, in inducing this disease; but it is even more remarkable that similar results should be obtained in regard to the relative prevalence and mortality by pleurisy and inflammation of the lungs, which are supposed to be still more influenced by these agencies. From a table it appears that inflammatory affections of the lungs are nearly twice as prevalent in the Mediterranean as among the same number of troops in the United Kingdom, and that in the mild climate of Malta they are also twice as fatal.

Here we must conclude our present notice of this valuable Report. North America has been yet unexplored, and to that region, replete as it now is with painful interest, we shall shortly turn.

We would direct attention, before we conclude, to this as well as to the previous Report on the West Indies. They will tend to disabuse the mind of many erroneous and some injurious impressions, and they will, we are confident, materially assist the progress of medical science.

